# Consistency of HFrEF treatment effect in underrepresented groups in randomized clinical trials

**DOI:** 10.1038/s44325-024-00028-4

**Published:** 2024-11-06

**Authors:** Guillaume Baudry, Luca Monzo, Mark C. Petrie, Nicolas Girerd, Ileana L. Piña, Alexandre Mebazaa, Javed Butler, Leila Abid, Faiez Zannad, Harriette G. C. Van Spall

**Affiliations:** 1 Université de Lorraine, Centre d’Investigations Cliniques Plurithématique, INSERM 1433, CHRU de Nancy, Institut Lorrain du Coeur et des Vaisseaux, Nancy, France; INI-CRCT (Cardiovascular and Renal Clinical Trialists) F-CRIN Network, Nancy, France; 2 REICATRA, Recherche et Enseignement en IC Avancée, Transplantation, Assistance, Vandœuvre-lès-Nancy, France; 3 MCP - School of Cardiovascular and Metabolic Health, University of Glasgow, Glasgow, Scotland; 4 Division of Cardiology, Thomas Jefferson University, Philadelphia, PA USA; 5 Université Paris Cité; MASCOT Inserm Unit; APHP, Paris, France; 6 Baylor Scott and White Research Institute, Dallas, TX and University of Mississippi, Jackson, MS USA; 7 Department of Cardiology, Hédi Chaker University Hospital, Faculty of Medicine of Sfax, University of Sfax, Sfax, Tunisia; 8 Department of Medicine, Faculty of Health Sciences, McMaster University, Hamilton, ON Canada; 9 Baim Institute for Clinical Research, Boston, MA USA

**Keywords:** Cardiology, Therapeutics, Heart failure

## Abstract

Despite the established efficacy of heart failure (HF) guideline-directed medical therapies, implementation varies across demographic groups, including Black, Indigenous, and people of color, older adults, females, and those who are socioeconomically deprived. This review synthesizes the representativeness of trial participants and describes subgroup analyses from pivotal trials in HF with reduced ejection fraction (HFrEF). It reviews the largely consistent treatment effect of medical therapies across the demographic groups represented in trials. It makes arguments for broad implementation of therapies based on these data, while calling for more representative trials to improve research and health equity in HF.

## Introduction

Heart failure (HF) is a leading cause of morbidity, mortality, and healthcare expenditure in adults across the world. With improved treatments for underlying causes such as myocardial infarction and for HF itself, the number of people living with HF continues to rise^1,2^. However, the benefits of treatments aren’t equally offered to all. Decades ago, the Board on Health Sciences Policy of the Institute of Medicine emphasized the need to include women and ethnic minorities in randomized clinical trials (RCT) for valid inferences about health and disease^3^. However, 40 years later, a majority of the global population with HF, including females, older adults, and ethnic minorities, are still under-represented in clinical trials^4^. There are relevant differences in access to or implementation of HF treatments across the world, with associated differences in HF survival^5^. In particular, older age, female sex, and race or ethnicity are factors associated with the under-use of guideline-directed medical therapies (GDMT) in registry or observational studies^6^.

**Fig. 1:**
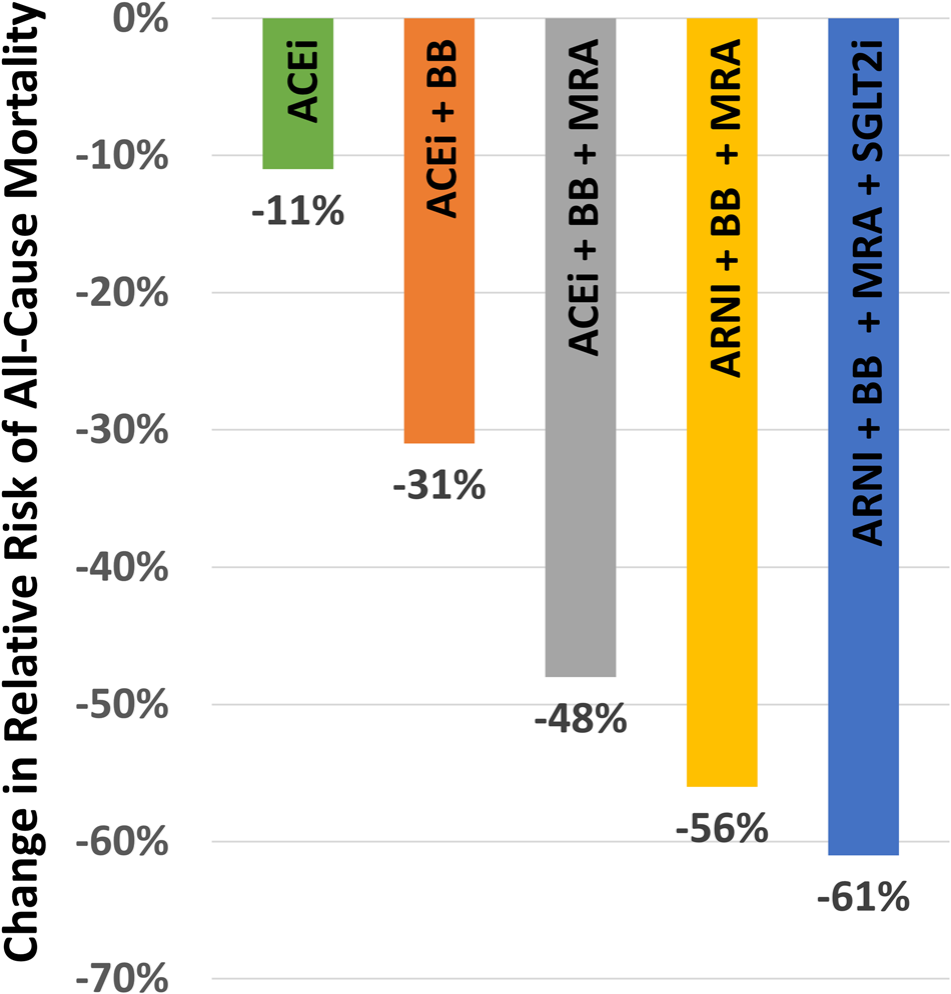
Cumulative treatment effect of Class-I recommended HF guideline-directed medical therapy classes on all-cause mortality. Estimates obtained from Tromp et al.^12^. ACEi Angiotensin-converting enzyme inhibitor, ARNI Angiotensin receptor-neprilysin inhibitor, BB Betablocker, MRA Mineralocorticoid receptor antagonist, SGLT2i Sodium glucose cotransporter-2 inhibitor. Change in relative risk of all-cause mortality with 95% confidence interval: ARNI + BB + MRA + SGLT2i: RRR = 0.61 (0.51–0.69). ARNI + BB + MRA: RRR = 0.56 (0.46–0.63). ACEI + BB + MRA: RRR = 0.48 (0.39–0.56). ACEI + BB: RRR = 0.31 (0.23–0.39). ACEI: RRR = 0.11 (0.04–0.18).

Based on high-quality RCT evidence, international guidelines recommend the use of betablockers, sodium glucose cotransporter-2 inhibitors (SGLT2i), mineralocorticoid receptor antagonists (MRAs), and renin-angiotensin system inhibitors (RASis) including angiotensin-converting enzyme inhibitors (ACEis), angiotensin receptor blockers (ARBs) or angiotensin receptor-neprilysin inhibitor (ARNI) in the treatment of HF with reduced ejection fraction (HFrEF) (Fig. 1)^7,8^. These four classes of GDMT have had suboptimal uptake in clinical settings, with variation across patient demographics^9,10^.

The implementation of GDMT faces limitations due to multi-level barriers. Concerns regarding the generalizability of clinical trial evidence- both efficacy and safety - are often cited as a reason for under-prescribing therapies^4,11^. Age, biological sex, and ethnicity can influence response to pharmacokinetics and pharmacodynamics, but under-represented groups in trials include older adults, females, and Black, Indigenous, and other people of color^4^. From a policy perspective, this gap in representation also has implications on cost-effectiveness and reimbursement decisions which rely on results from RCTs. The objective of this review is to synthesize the treatment effect of Class I and II recommended GDMTs in groups that were underrepresented in pivotal HFrEF RCTs (Fig. 1). These RCTs have enrolled over 100,000 patients with follow-up spanning more than 200,000 patient-years^12^. These invaluable data can provide insights into treatment effect in these subgroups, while also highlighting knowledge gaps that we must close to undertake meaningful subgroup analyses, which require adequate sample sizes in each subgroup to provide precise effect estimates and test for treatment interactions. In this integrative review, we included data from multicenter pivotal RCTs of GDMT evaluating the effects of treatments compared to placebo or standard treatment, without any date limitation until June 2023. A pivotal trial was defined as a large-scale, multicenter trial that informed the Class I recommendation of a particular treatment class in European or American Heart Failure guidelines.

## Racial and ethnic diversity

**Fig. 2:**
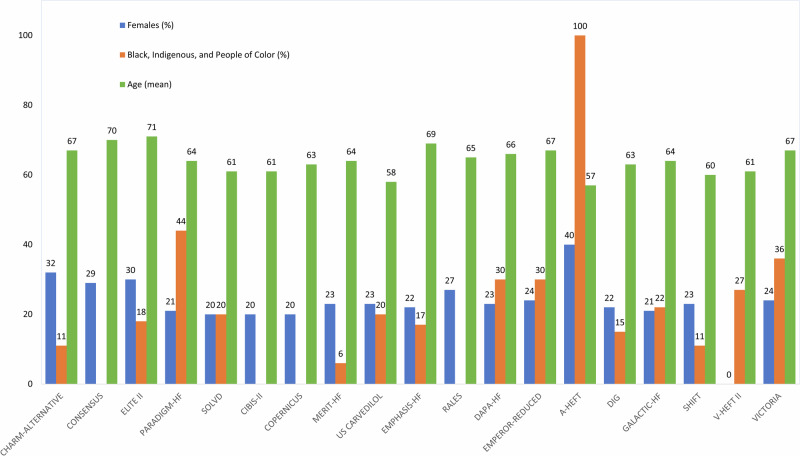
Mean age and demographics of participants in pivotal HF randomized clinical trials. A-HeFT African-American Heart Failure Trial, CIBIS-II Cardiac Insufficiency Bisoprolol Study II, CHARM-Alternative Candesartan in Heart failure: Assessment of Reduction in Mortality and morbidity (Alternative), CONSENSUS Cooperative North Scandinavian Enalapril Survival Study, COPERNICUS Carvedilol Prospective Randomized Cumulative Survival, DAPA-HF dapagliflozin and prevention of adverse outcomes in Heart Failure, DIG Digitalis Investigation Group, ELITE II Evaluation of Losartan In The Elderly II, EMPEROR-REDUCED Empagliflozin Outcome Trial in Patients with Chronic Heart Failure and a Reduced Ejection Fraction, EMPHASIS-HF Eplerenone in Mild Patients Hospitalization and Survival Study in Heart Failure, GALACTIC-HF Global Approach to Lowering Adverse Cardiac Outcomes through Improving Contractility in Heart Failure, MERIT-HF Metoprolol CR/XL Randomized Intervention Trial in Congestive Heart Failure, PARADIGM-HF Prospective Comparison of ARNI with ACEI to Determine Impact on Global Mortality and Morbidity in Heart Failure, RALES Randomized Aldactone Evaluation Study, SHIFT Systolic Heart failure treatment with the If inhibitor ivabradine Trial, SOLVD Studies Of Left Ventricular Dysfunction, US Carvedilol U.S. Carvedilol Heart Failure Study, V-HeFT II Vasodilator–Heart Failure Trial II, VICTORIA Vericiguat Global Study in Subjects with Heart Failure with Reduced Ejection Fraction.

The Food and Drug Administration, prompted by potential ethnic-related variations in the effectiveness and tolerance of HF drugs, has appropriately highlighted the importance of representativeness in clinical trials, urging trialists to collect demographic data and participants to self-report their race or ethnicity^13,14^. The reporting of race-related information in RCTs is suboptimal, with missed reporting in a majority of trials and limited reporting in others. Presently, race information is accessible in fewer than 40% of HF RCTs published in high impact journals, with BIPOC (Black, Indigenous, and People of Color) individuals comprising less than 20% of participants, despite a gradual increase observed from 2000 to 2020 (Fig. 2)^15^. Moreover, the trial classification of race or ethnicity has changed over time. Initially, trial descriptions of race were limited to three categories (White, Black, and other), but later expanded to four categories (White, Black, Asian, and other), with recognition that further disaggregation may be warranted based on differences in ancestry and disease phenotypes. In addition to possible ancestral differences, race or ethnicity entails socio-cultural differences, leading to variations in behavior, diet, and access to healthcare, which can influence treatment effect. Therefore, evaluating treatment effects across ethnic subgroups should be approached with nuance when interpreting the results.

Data are lacking on the effects of historic drugs, such as ACEis and betablockers, in specific subgroups, such as South or East Asian patients. Notably, ethnicities like North African or Arab or Hispanic Black are traditionally not reported in RCTs, leading to a gap in knowledge regarding the effects of medical interventions in these populations. Trials embedded in registries or administrative datasets are limited to the race or ethnicity data held in these datasets and many jurisdictions (e.g. in France and Canada) have policies that restrict the collection and use of such data for perceived ethical or privacy reasons.

There are conflicting data on treatment heterogeneity in the effect of ACEis across race groups, and no evidence of race-treatment interaction in the effect of ARNI (Table 1). The initial observations of race as an effect modifier of ACEis in HF originated from V-HeFT II (Vasodilator-Heart Failure trial II) (Table 5), which compared the efficacy of hydralazine plus isosorbide dinitrate versus enalapril in treating chronic HF^16^. Mortality reduction with enalapril was noted in White participants, while the results were inconclusive in Black participants, without a significant interaction (p for interaction = 0.09)^17^. The SOLVD (Studies of Left Ventricular Dysfunction) prevention and treatment studies offer insights into the benefits of ACEis within the Black subgroup. The first post-hoc analysis of pooled data from these studies, involving 800 Black patients and 1196 White patients, demonstrated that enalapril was associated with a smaller reduction in HF hospitalization among Black (14%) than White (49%) patients (race-treatment interaction p-value = 0.005)^18,19^. In the second post-hoc analysis, limited to the SOLVD prevention trial with 3651 White and 403 Black patients, there was no significant race-treatment interaction with enalapril, thus leaving the question unresolved^19^. A recent individual patient data meta-analysis^20^ of five RCTs on the effect of RAS inhibitors in heart failure, including SOLVD-Prevention, SOLVD-Treatment, CHARM-Alternative, CHARM-Added, and the Valsartan Heart Failure Trial (Val-HeFT)^21^, was conducted. The hazard ratio (HR) for RAS blockade versus placebo for the primary composite outcome of first hospitalization for heart failure or cardiovascular death was 0.84 (95% CI, 0.69–1.03) in Black patients and 0.73 (95% CI, 0.67-0.79) in non-Black patients (P for interaction = 0.14)^20^. The corresponding HRs for cardiovascular death were 0.83 (95% CI, 0.65-1.07) and 0.84 (95% CI, 0.77-0.93), respectively (P for interaction = 0.99)^20^. In the PARADIGM-HF trial (Table 1) sacubitril-valsartan decreased the primary end point of death from cardiovascular causes or HF hospitalization, with no evidence of a race-treatment interaction (interaction p-value of 0.58)^22^.

**Table 1:** Treatment effect of renin-angiotensin system inhibitors across demographic groups in pivotal HFrEF randomized clinical trials

Study name	CHARM-alternative	CONSENSUS	ELITE II	PARADIGM-HF	SOLVD
Target population	Chronic HF, LVEF ≤ 40%, NYHA class II to IV, not receiving ACEis because of previous intolerance	NYHA IV	Chronic HF, LVEF ≤ 40%, NYHA class II to IV	Chronic HF, LVEF ≤ 40%, NYHA class II to IV	Chronic HF, LVEF ≤ 35%
Intervention	Candesartan vs placebo	Enalapril. vs placebo	Losartan vs captopril	LCZ696 vs enalapril	Enalapril vs placebo
Number of patients	2028	253	3152	8442	2569
Location/region	618 centers in 26 countries in Africa, Europe, North America and Oceania	35 centers in Europe	289 centers in 46 countries	1043 centers in 47 countries in Africa, Asia, Europe, North America, South America	23 centers in Europe and North America
Year	2003	1987	2000	2014	1991
Females (%)	32	29	30	21	20
Age (mean)	67	70	71	64	61
BIPOC (%)	11	NA	18	44	20
Older adults (aged >75 years)	23%	NA	NA	19%	NA
Primary endpoint	Composite of death from cardiovascular causes or hospitalization for heart failure	All-cause mortality	All-cause mortality	Composite of death from cardiovascular causes or hospitalization for heart failure	All-cause mortality
Treatment effect	HR 0.77 (95% CI 0.67–0.89), *p* = 0.0004	RRR 0.40, *p* = 0.002	HR 1.13 (95% CI 0.95–1.35), *p* = 0.16	HR 0.80 (95% CI 0.73–0.87), *P* < 0.001	RRR 0.84 (95% CI 0.05–0.26), *p* = 0.004
Effect in sex subgroups	NA	NA	Female 1.14 Male 1.12	LCZ696 better / no heterogeneity in sex subgroups	NA
P-value, sex-treatment interaction	NA	NA	NS	*p* = 0.63	NA
Effect in race or ethnicity subgroups	NA	NA	NA	LCZ696 better/no heterogeneity in color subgroups	NA
*P*-value, race or ethnicity-treatment interaction	NA	NA	NA	0.58	NA
Effect in age subgroups	NA	NA	<70 yr 1.33 ≥70 yr 1.05	(65 yr) LCZ696 better/no heterogeneity (75 yr) LCZ696 better/no heterogeneity	NA
*P*-value, age-treatment interaction	NA	NA	NA	(65 yr) *p* = 0.47 (75 yr) *p* = 0.32	NA
Effect in socioeconomic subgroups	NA	NA	NA	NA	NA

*BIPOC* Black, Indigenous, and People of Color, *CHARM-Alternative* Candesartan in Heart failure: Assessment of Reduction in Mortality and morbidity (Alternative), *CONSENSUS* Cooperative North Scandinavian Enalapril Survival Study, *ELITE II* Evaluation of Losartan In The Elderly II, *HR* Hazard ratio, *NA* Not available, *NS* Non-significant based on *p*-value of >0.05 (exact value was not provided), *PARADIGM-HF* Prospective Comparison of ARNI with ACEI to Determine Impact on Global Mortality and Morbidity in Heart Failure, *RRR* relative risk reduction, *SOLVD* Studies Of Left Ventricular Dysfunction, *vs* versus.

Currently, there is no evidence to support an interaction between race or ethnicity and the treatment effect of betablockers. A post-hoc analysis of the U.S. CARVEDILOL HF Trials assessed the treatment effect of carvedilol according to race. The reduction in the composite of death or HF hospitalization was similar in Black versus other patients, with no significant race-treatment interaction (*p* for interaction = 0.78)^23^.

SGLT2is have demonstrated a consistent effect in reducing cardiovascular death or worsening HF across race groups, although there may be a larger treatment effect in Black relative to White patients, with some of these differences possibly related to different regionality. Indeed, subgroup analysis of pooled data from DAPA-HF and EMPEROR-REDUCED showed a benefit of SGLT2is across all ethnicities, but with a greater reduction in the primary composite endpoint of cardiovascular death or worsening HF in Black (47%) and Asian (39%) compared with White (17%) patients, with a significant race interaction (p for interaction =0.0063)^24^. These finding were evident in the underlying trial, with EMPEROR-REDUCED depicting a stronger reduction in the composite endpoint of cardiovascular death or worsening HF among Black (51%) and Asian (52%) in comparison to White (19%) patients (p for interaction = 0.002)^25^. The DAPA-HF study highlighted the interplay between race and geographic regions. The more pronounced effect among Black individuals in the overall population was driven by differences in regions outside the Americas, and the reduction of the combined endpoint of cardiovascular death or worsening HF in patients enrolled in the Americas showed no significant differences according to race (38% in Black patients vs. 32% in White patients, interaction p-value = 0.7)^26^.

In conclusion, there is no evidence of heterogeneity in treatment effect to justify varied use of GDMT based on race or ethnicity. The interplay between race and geographic location warrants further analysis in future therapeutic trials.

## Age

HF predominantly affects older individuals, with its incidence escalating with age — from approximately 7 per 1000 person-years after 45 years of age to around 21 per 1000 population after 65 years of age. Similarly, HF prevalence increases with age from under 3% in subjects younger than 60 years old to 9% in individuals over 80 years of age^27,28^. While older adults are at higher risk of worsening heart failure events, they are less likely to receive optimal doses of GDMT even after adjusting for other factor^29,30^.

Despite adults over 70 years comprising 62.2% of global HF cases, amounting to approximately 34.4 million individuals, RCTs predominantly focus on a younger HF demographic, with a mean age between 60 and 70 years (Tables 1 to 5 and Fig. 2). This discrepancy arises because clinical trials often exclude individuals with comorbidities such as cognitive dysfunction, kidney dysfunction, lung disease, or perceived frailty. Current HF treatment guidelines do not specifically tailor recommendations for older patients^8^.

**Table 2:** Treatment effect of betablockers across demographic groups in pivotal HFrEF randomized clinical trials

Study name	CIBIS-II	COPERNICUS	MERIT-HF	US Carvedilol
Target population	Chronic HF, LVEF ≤ 35%, NYHA class III or IV	Chronic HF, LVEF ≤ 25%, NYHA class III or IV	Chronic HF, LVEF ≤ 40%, NYHA class II to IV	Chronic HF, LVEF ≤ ≤ 35%
Intervention	Bisoprolol vs placebo	Carvedilol vs placebo	Metoprolol vs placebo	Carvedilol vs placebo
Number of patients	2647	2289	3991	1094
Location/region	274 hospitals in 18 countries in Europe	334 centers in 21 countries in Africa, Asia, Europe, North America and Oceania	313 centers in 13 countries in Europe and North America	65 participating centers in North America
Year	1999	2001	1999	1996
Females (%)	20	20	23	23
Age (mean)	61	63	64	58
BIPOC (%)	NA	NA	6	20
Older adults (aged >75 years)	Age >80, 25%	NA	Age >70, 10%	NA
Primary endpoint	All-cause mortality	All-cause mortality	All-cause mortality	All-cause mortality or hospitalization for cardiovascular reasons
Treatment effect	HR 0.66 (95% CI 0.54–0.81), *p* < 0.0001	RRR 0.35 (95% CI 0.19–0.48), *p* = 0.0014	HR 0.66 (95% CI 0.53–0.81), *p* < 0.0001	RR 0.65 (95% CI 0.39–0.80), *p* < 0.001
Effect in sex subgroups	Female 0.52 Male 0.71	Female 0.65 Male 0.65	Female 0.92 Male 0.61	Female 0.23 (0.07–0.69)* Male 0.41 (0.22–0.80)*
*P*-value, sex-treatment interaction	NS	NS	*p* = 0.14	NS
Effect in race or ethnicity subgroups	NA	NA	NA	Black patients 0.68 (0.37–1.23) Other patients 0.65 (0.48–0.88)
*P*-value, race or ethnicity-treatment interaction	NA	NA	NA	*p* = 0.89
Effect in age subgroups	NA	(65 yr) Carvedilol better /No heterogeneity	(69 yr) metoprolol better/No heterogeneity	<59 yr 0.30 (0.11–0.80)* ≥59 yr 0.38 (0.19–0.77)*
*P*-value, age-treatment interaction	NA	NS	NS	NS
Effect in socioeconomic subgroups	NA	NA	NA	NA

*BIPOC* Black, Indigenous, and People of Color, *CIBIS-II* Cardiac Insufficiency Bisoprolol Study II, *COPERNICUS* Carvedilol Prospective Randomized Cumulative Survival, *HR* Hazard ratio, *MERIT-HF* Metoprolol CR/XL Randomized Intervention Trial in Congestive Heart Failure, *NA* Not available, *NS* Non-significant based on *p*-value of >0.05 (exact value was not provided), *RRR* relative risk reduction, *US Carvedilol* U.S. Carvedilol Heart Failure Study, *vs* versus.

*Reported only for all-cause mortality.

**Table 3:** Treatment effect of mineralocorticoid receptor antagonists across demographic groups in pivotal HFrEF randomized clinical trials

Study name	EMPHASIS-HF	RALES
Target population	Chronic HF, LVEF ≤ 35%, NYHA class II	Chronic HF, LVEF ≤ 35%, NYHA class III or IV
Intervention	Eplerenone vs placebo	Spironolactone vs placebo
Number of patients	2737	1663
Location/region	278 centers in 29 countries in Africa, Asia, Europe, North America, Oceania and South America	195 centers in 15 countries in Africa, Asia, Europe, North America, Oceania and South America
Year	2011	1999
Females (%)	22	27
Age (mean)	69	65
BIPOC (%)	17	NA
Older adults (aged >75 years)	Age >75, 24%	NA
Primary endpoint	Composite of death from cardiovascular causes or hospitalization for heart failure	All-cause mortality
Treatment effect	HR 0.63 (95% CI 0.54–0.74), *p* < 0.001	HR 0.70 (95% CI 0.60–0.82), *p* < 0.001
Effect in sex subgroups	Eplerenone better /No heterogeneity	Eplerenone better /No heterogeneity
*P*-value, sex-treatment interaction	*p* = 0.36	NS
Effect in race or ethnicity subgroups	NA	NA
*P*-value, race or ethnicity-treatment interaction	NA	NA
Effect in age subgroups	(65 yr) Eplerenone better /No heterogeneity (75 yr) Eplerenone better/No heterogeneity	(67 yr) Spironolactone better/No heterogeneity
*P*-value, age-treatment interaction	(65 yr) *p* = 0.37 (75 yr) *p* = 1.00	(67 yr) NS
Effect in socioeconomic subgroups	NA	NA

*BIPOC* Black, Indigenous, and People of Color, *EMPHASIS-HF* Eplerenone in Mild Patients Hospitalization and Survival Study in Heart Failure, *HR* Hazard ratio, *NA* Not available, *NS* Non-significant based on *p*-value of >0.05 (exact value was not provided), *RALES* Randomized Aldactone Evaluation Study, *vs* versus.

**Table 4:** Treatment effect of sodium glucose cotransporter-2 inhibitors across demographic groups in pivotal HFrEF randomized clinical trials

Study name	DAPA-HF	Emperor-reduced
Target population	Chronic HF, LVEF ≤ 40%, NYHA class II to IV	Chronic HF, LVEF ≤ 40%, NYHA class II to IV
Intervention	Dapagliflozin vs placebo	Empagliflozin vs placebo
Number of patients	4744	3730
Location/region	410 centers in 20 countries in Africa, Asia, Europe, North America, and Oceania	520 centers in 20 countries in Asia, Europe, North America, Oceania, and South America
Year	2019	2020
Females (%)	23	24
Age (mean)	66	67
BIPOC (%)	30	30
Older adults (aged >75 years)	24%	27%
Primary endpoint	Composite of worsening heart failure (hospitalization or an urgent visit) or cardiovascular death	Composite of cardiovascular death or hospitalization for worsening heart failure
Treatment effect	HR 0.74 (95% CI 0.65–0.85), *p* < 0.001	HR 0.75 (95% CI 0.65–0.86), *p* < 0.001
Effect in sex subgroups	Female 0.79 (0.59–1.06) Male 0.73 (0.63–0.85)	Female 0.59 (0.44–0.80) Male 0.80 (0.68–0.93)
*P*-value, sex-treatment interaction	*p* = 0.67	NS
Effect in race or ethnicity subgroups	White 0.78 (0.66–0.91) Black 0.62 (0.37–1.04) Asian 0.64 (0.48–0.86) Other NA	White 0.88 (0.75, 1.04) Black 0.46 (0.28, 0.75) Asian 0.57 (0.41, 0.78) Other 0.41 (0.15, 1.14)
*P*-value, race or ethnicity-treatment interaction	*p* = 0.70	*p* = 0.008
Effect in age subgroups	<55 yr 0.87 (0.60–1.28) 55–64 yr 0.71 (0.55–0.93) 65–74 yr 0.76 (0.61–0.95) ≥75 y 0.68 (0.53–0.88)	<65 yr 0.71 (0.57, 0.89) 65 to <75 yr 0.72 (0.57, 0.93) ≥75 yr 0.86 (0.67, 1.10)
*P*-value, age-treatment interaction	*p* = 0.76	*p* = 0.24
Effect in socioeconomic subgroups	NA	NA

*BIPOC* Black, Indigenous, and People of Color, *DAPA-HF* dapagliflozin and prevention of adverse outcomes in Heart Failure, *EMPEROR-REDUCED* Empagliflozin Outcome Trial in Patients with Chronic Heart Failure and a Reduced Ejection Fraction, *HR* Hazard ratio, *NA* Not available, *NS* Non-significant based on *p*-value of >0.05 (exact value was not provided), *vs* versus.

In most post hoc analyses of RCTs assessing age-treatment interaction, a consistent benefit has been observed in older patients^31–35^. A comprehensive meta-analysis in patients with HFrEF found that betablockers led to a similar reduction in mortality across all age groups (HR 0.65–0.77) without significant age interaction (*p* for interaction = 0.1)^35^. The SENIORS trial, specifically designed to evaluate nebivolol’s effects in older HFrEF patients (inclusion criteria: age ≥70 years; mean age 76 years), demonstrated a significant reduction in the composite of all-cause mortality or cardiovascular hospital admission in the intervention group without age interaction (*p* for interaction = 0.51)^36^.

Similarly, in the PARADIGM-HF trial the benefit of sacubitril/valsartan vs. enalapril was consistent in all age categories (i.e. <55 years, 55–64 years, 65–74 years, and ≥75 years)^33^.

In a recent secondary analysis of the STRONG-HF (safety, tolerability, and efficacy of up-titration of guideline-directed medical therapies for acute HF) RCT, researchers found that the benefits of up-titrating beta-blockers, RASi, and MRA following hospitalization for HF were similar in both older (>65 years) and younger patients, showing no significant interaction between treatment and age (interaction *p* = 0.57)^37^. Additionally, the incidence of adverse effects was consistent across both age groups^37^.

A recent meta-analysis demonstrated that SGLT2is uniformly benefit cardiovascular outcomes in people ≤65 and >65 years of age, as well as specific age subgroups (<55 years, 55–64 years, 65–74 years, and ≥75 years)^24^. Further, post-hoc analyses of the DAPA-HF and EMPEROR-Reduced trials confirmed that treatments with dapagliflozin and empagliflozin have consistent beneficial treatment effects across age groups on primary outcomes and on kidney function renal function decline, with no differences in serious side effects across age groups^31,34^.

No significant age-treatment interaction was observed in recent trials testing the efficacy of vericiguat and omecamtiv mecarbil, as indicated in Table 5^38,39^.

In summary, GDMT seems effective across age categories of adults enrolled in HF RCT. The inclusion of older adults in RCTs is vital, particularly as the HF prevalence rises with aging populations.

## Sex

Females are underrepresented in HF clinical trials^40^, despite recommendations for their inclusion by the National Institutes of Health Revitalization Act in 1992^41^, the US Food and Drug Administration guideline in 1993^42^, the European Medicines Agency guideline in 2005^43^, and the Canadian Institutes of Health Research guideline in 2010^44^. Indeed, in HFrEF RCTs, females represent approximately less than 25% of the total study participants, with prevalence being lower in interventional trials and marginally higher in trials for HF with preserved ejection fraction (HFpEF) (Tables 1–5 and Fig. 2)^45,46^. These sex differences in enrollment have remained unchanged over the past two decades, and exclusion of women who have childbearing potential from pivotal HF GDMT trials remains standard practice^40^.

The underlying reasons for sex imbalance in HF clinical trials relative to disease distribution are multifaceted, spanning across trial design, clinician, and patient. Trial factors independently associated with under-representation include sex specific exclusion criteria related to the potential for pregnancy (not routinely justified in the context of the intervention)^47,48^, recruitment setting, and men-only trial leadership team^40^. Females may also be more likely to meet trial exclusion criteria due to factors like older age, cognitive dysfunction, frailty, or multimorbidities^53^. The role of referral biases or patient consent are less clear^40,49–51^.

The effectiveness of RASis is consistent between sexes (Table 1), with some evidence of a sex-LVEF-treatment interaction such that ARB, ARNI, and MRA were beneficial to a higher range of LVEF in females than males (outside the scope of this review)^21,52–54^. A meta-analysis of individual patient data, which included 12,763 patients from five RCTs, three of which were post-infarction, found a consistent mortality reduction in males (OR 0.79, 95% CI 0.72–0.87) and females (OR 0.85, 95% CI 0.71–1.02), with no sex-treatment interaction (*p* for interaction 0.54)^52^.

Individual analyses of RCTs assessing the effectiveness of betablockers in HFrEF (US Carvedilol, CIBIS-II, MERIT-HF and COPERNICUS) consistently show a consistent reduction in all-cause mortality in males and females, with no significant sex differences (Table 2). This observation is further supported by a meta-analysis that included 11 RCTs with over 13,800 HFrEF patients and demonstrated that a similar mortality benefit of betablockers across sex groups^35^.

The SGLT2is dapagliflozin and empagliflozin demonstrated a similar reduction of cardiovascular mortality and HF hospitalization in both sexes in HFrEF, without sex-treatment interaction (Table 4)^22,24^. Specifically, dapagliflozin demonstrates a consistent benefit in reducing primary outcome components as well as enhancing quality of life in both male and female patients^55^. In addition, when combined together, recommended drugs for HFrEF provide a significant risk reduction in cardiovascular events, irrespective of sex^56^.

Among second-line HFrEF therapies, digoxin exhibited a significant sex-specific interaction in the Digitalis study (*p* for interaction = 0.034)^57^. Digoxin had a harmful effect in females, with an increased mortality risk difference (RD) of 4.2% (95% CI: −0.5 to 8.8) relative to placebo; this was in comparison to a RD of −1.6% in males (95% CI: −4.2 to 1.0), as shown in Table 5^57^.

Recent data indicate that for females, 60% of the recommended dose of beta-blockers or RAAS inhibitors may be sufficient to reduce the risk of all-cause death or HF hospitalization by 30%, whereas a higher dose may be necessary in males^58,59^.

Finally, the recent findings from the STRONG-HF trial following hospitalization for HF showed a comparable benefit of a rapid GDMT up-titration strategy after an acute HF hospitalization in both males and females^60^. Similarly, no sex-based interaction was observed with vericiguat (*p* for interaction = 0.79) or omecamtiv mecarbil treatments (*p* for interaction = 0.68), as shown in Table 5^38,39,61^.

In conclusion, there appears to be consistent treatment benefit across the Class I recommended GDMT in males and females, with evidence of efficacy of RASi and MRA at a higher LVEF range in females than males based on post-hoc meta-analysis. A signal for a possible differential sex effect with harm to females seems to be present for digoxin. The effect of these therapies in pregnant or lactating females has never been tested due to the categorical exclusion of pregnant and lactating females from HF RCTs.

## Socioeconomic status and education level

Low socioeconomic status and education level confer an independent risk for incident HF and are associated with unfavorable outcome^62,63^ partly related to a higher prevalence of cardiovascular risk factors, decreased physical activity, environmental and nutritional factors, treatment costs, and disparities in healthcare^64,65^.

In the recent CHAMP-HF Registry from United States, socioeconomic status did not show any association with medication use or treatment dosages^66^. In a meta-analysis including 28 studies (no RCTs) mainly conducted in western countries (1 study from Brazil), socioeconomic status was associated with incidence and prevalence of HF, risk of HF readmission and all-cause mortality^70^. These data were confirmed in a population-based study from United Kingdom and in the Swedish HF Registry^67,68^. Notably, individuals with lower socioeconomic status face 9–22% higher risks of morbidity and mortality, irrespective of HF severity and quality of care^67^.

Information on social and educational levels is rarely available in RCTs, preventing the assessment of potential interactions with treatment and importantly, the correct interpretation of any treatment differences attributed to age, sex or gender, and race or ethnicity^69^. Socioeconomic status varies across age, race and gender groups, and provides important context that can guide inferences regarding treatment heterogeneity across any one of these groups. While there is no reason to believe that the biological effect of medications varies across socioeconomic strata, other factors such as adherence and access to healthcare could be influenced by socioeconomic status and potentially interact with treatment efficacy. Yet, socioeconomic remains unexplored as a subgroup in assessing treatment effect in pivotal HF trials (Tables 1–5).

**Table 5:** Treatment effect of second-line heart failure medications across demographic groups in pivotal HFrEF randomized clinical trials

Study name	A-HeFT	DIG	GALACTIC-HF	SHIFT	V-HeFT II	VICTORIA
Target population	Chronic HF, NYHA class III to IV	Chronic HF, LVEF ≤ 45%,	Chronic HF, LVEF ≤ 35%, NYHA class II to IV	Chronic HF, LVEF ≤ 40%, NYHA class II to IV, in sinus rhythm & heart rate ≥70 bpm	Chronic HF	Chronic HF, LVEF ≤ 45%, NYHA class II to IV
Intervention	Isosorbide dinitrate plus hydralazine vs placebo	Digoxin vs placebo	Omecamtiv mecarbil vs placebo	Ivabradine vs placebo	Enalapril vs hydralazine plus isosorbide dinitrate	Vericiguat vs placebo
Number of patients	1050	6800	8256	6558	804	5050
Location/region	161 centers in North America	302 centers in 2 countries in North America	945 centers in 35 participating countries in Africa, Asia, Europe, North America, Oceania, and South America	677 centers in 37 countries in Asia, Europe, North America, Oceania, and South America	13 centers in North America	616 centers in 42 countries in Asia, Europe, North America, Oceania, and South America
Year	2004	1997	2021	2010	1991	2020
Females (%)	40	22	21	23	0	24
Age (mean)	57	63	64	60	61	67
BIPOC (%)	100	15	22	11	27	36
Older adults (aged >75 years)	NA	Age > 70 yr, 27%	NA	Age ≥ 69 yr, 26%	NA	Age ≥ 75 yr, 31%
Primary endpoint	Composite score made up of weighted values for death from any cause, a first hospitalization for heart failure, and change in the quality of life	All-cause mortality	Composite of worsening heart failure (hospitalization or an urgent visit) or cardiovascular death	Composite of cardiovascular death or hospital admission for worsening heart failure.	All-cause mortality at 2 years	Death from cardiovascular causes or first hospitalization for heart failure
Treatment effect	−0.1 ± 1.9 vs. −0.5 ± 2.0, *P* = 0.01	HR 0.99 (95% CI 0.91–1.07) *p* = 0.80	HR 0.92 (95% CI 0.86 to 0.99) *p* = 0.03	HR 0.82 (95% CI 0.75–0.90), *p* < 0.0001	RRR 0.28, *p* = 0.016	HR 0.90 (95% CI 0.82–0.98), *p* = 0.02
Effect in sex subgroups	NA	Female RD 4.2 (95% CI −0.5 to 8.8) Male RD −1.6% (95% CI −4.2 to 1.0)	Female 0.95 (0.81–1.12) Male 0.92 (0.85–0.99)	Female 0.74 (0.60–0.91) Male 0.84 (0.76–0.94)	–	Female 0.88 (0.73–1.08) Male 0.90 (0.81–1.00)
*P*-value, sex-treatment interaction	NA	*p* = 0.034	*p* = 0.68	*p* = 0.260	–	*p* = 0.788
Effect in race or ethnicity subgroups	–	NA	White 0.95 (0.88–1.03) Black 0.82 (0.64–1.04) Asian 0.79 (0.61–1.02) Other 0.91 (0.69–1.21)	NA	NA	White 0.91 (0.81, 1.02) Black 0.85 (0.56, 1.28) Asian 0.91 (0.75, 1.11) Other 0.80 (0.57, 1.11)
*P*-value, race or ethnicity-treatment interaction	–	NS	NS	*p* = 0.5	*p* = 0.09	NS
Effect in age subgroups	NA	NA	<65 yr 0.91 (0.82–1.02) ≥65 yr 0.94 (0.86–1.03)	<65 yr 0.76 (0.67–0.87) ≥65 yr 0.89 (0.77–1.02)	NA	<65 yr 0.81 (0.70, 0.95) ≥65 yr 0.94 (0.84–1.06) <75 yr 0.84 (0.75, 0.94) ≥75 yr 1.04 (0.88–1.21)
*P*-value, age-treatment interaction	NA	*p* = 0.55	NS	*p* = 0.099	NA	Age continuous *p* = 0.169 Age categorical ( < 65 yr, 65–74 yr, ≥75 yr) *p* = 0.189
Effect in socioeconomic subgroups	NA	NA	NA	NA	NA	NA

*A-HeFT* African-American Heart Failure Trial, *BIPOC* Black, Indigenous, and People of Color, *DIG* Digitalis Investigation Group, *GALACTIC-HF* Global Approach to Lowering Adverse Cardiac Outcomes through Improving Contractility in Heart Failure, *HR* Hazard ratio, *NA* Not available, *NS* Non-significant based on *p*-value of >0.05 (exact value was not provided), *RRR* relative risk reduction, *SHIFT* Systolic Heart failure treatment with the If inhibitor ivabradine Trial, *V-HeFT II* Vasodilator–Heart Failure Trial II, *VICTORIA* Vericiguat Global Study in Subjects with Heart Failure with Reduced Ejection Fraction, *vs* versus.

**Fig. 3:**
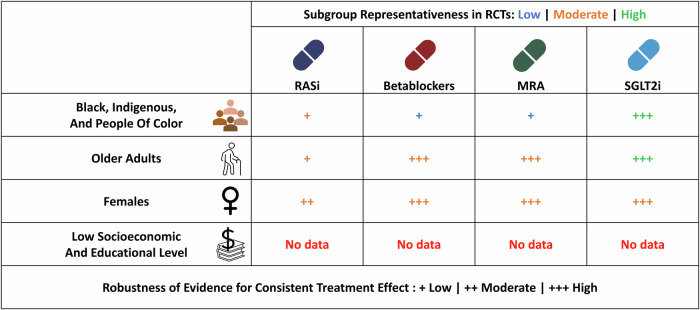
Consistency of heart failure treatment effect in underrepresented subgroups. HF Heart failure, GDMT Guideline directed medical therapy, MRA Mineralocorticoid receptor antagonist, RASi Renin-angiotensin system inhibitor, RCT Randomized clinical trial, SGLT2i Sodium glucose cotransporter-2 inhibitor. This figure illustrates the representativeness (low in red, moderate in orange, and high in green) of various subgroups in randomized clinical trials for Class I recommended heart failure therapies, as well as the robustness of evidence regarding the consistency of treatment effects. While current literature does not show significant heterogeneity in the treatment effects of Class I recommended therapies, this absence of heterogeneity may either indicate a true lack of variation or a lack of statistical power to detect it. The number of crosses in the figure reflects the robustness (low +, moderate ++, and high + + +) of the data supporting the absence of heterogeneity, with one cross indicating limited available data and three crosses representing strong, reliable data confirming the absence of heterogeneity.

The Fig. 3 illustrates the representativeness (low in red, moderate in orange and high in green) and robustness of evidence regarding treatment consistency (low +, moderate ++ and high +++) in underrepresented subgroups for the four first-line therapeutic classes of HF based on data from pivotal RCTs.

In conclusion, HF patients from underrepresented groups, including BIPOC, females, and older adults are under-represented in RCTs and often receive suboptimal medical treatment in clinical settings. Testing for and reporting treatment heterogeneity in these groups have varied with time. In addition, under-representation has been a limitation in conducting meaningful subgroup analysis. Subgroups that are markedly imbalanced may lack the statistical power required to detect treatment heterogeneity. The limitations of testing for interaction are also worth considering. Despite these limitations, evidence from more contemporary trials does not suggest differences in the efficacy of Class I recommended GDMT in HFrEF across explored demographic groups. Some differences attributed to race may be related to region, as demonstrated in a trial of SGLT2i, and the role of socioeconomic status on outcomes across GDMT classes is unknown across all pivotal trials. Because safety events are not reported by subgroups, the tolerability of GDMT across demographic groups remains unclear and relies on careful monitoring in clinical settings, especially during treatment up-titration. While it is important to continue to aim for representativeness in clinical trials, it is equally important to implement effective therapies in all people living with HF unless there is evidence for heterogeneity in treatment effect.

## Data Availability

No datasets were generated or analysed during the current study.
