# Author Correction: Consistency of HFrEF treatment effect in underrepresented groups in randomized clinical trials

**DOI:** 10.1038/s44325-025-00045-x

**Published:** 2025-02-03

**Authors:** Guillaume Baudry, Luca Monzo, Mark C. Petrie, Nicolas Girerd, Ileana L. Piña, Alexandre Mebazaa, Javed Butler, Leila Abid, Faiez Zannad, Harriette G. C. Van Spall

**Affiliations:** 1 Université de Lorraine, Centre d’Investigations Cliniques Plurithématique, INSERM 1433, CHRU de Nancy, Institut Lorrain du Coeur et des Vaisseaux, Nancy, France; INI-CRCT (Cardiovascular and Renal Clinical Trialists) F-CRIN Network, Nancy, France; 2 REICATRA, Recherche et Enseignement en IC Avancée, Transplantation, Assistance, Vandœuvre-lès-Nancy, France; 3 MCP - School of Cardiovascular and Metabolic Health, University of Glasgow, Glasgow, Scotland; 4 Division of Cardiology, Thomas Jefferson University, Philadelphia, PA USA; 5 Université Paris Cité; MASCOT Inserm Unit; APHP, Paris, France; 6 Baylor Scott and White Research Institute, Dallas, TX and University of Mississippi, Jackson, MS USA; 7 Department of Cardiology, Hédi Chaker University Hospital, Faculty of Medicine of Sfax, University of Sfax, Sfax, Tunisia; 8 Department of Medicine, Faculty of Health Sciences, McMaster University, Hamilton, ON Canada; 9 Baim Institute for Clinical Research, Boston, MA USA

**Keywords:** Cardiology, Therapeutics, Heart failure

Correction to: *npj Cardiovascular Health* 10.1038/s44325-024-00028-4, published online 06 November 2024

In the original Article, a sentence was missing from the abstract. The missing sentence has now been added to the abstract and the original Article has been corrected.

